# Outcomes of 23-Gauge Pars Plana Vitrectomy in Various Vitreoretinal Diseases

**DOI:** 10.7759/cureus.72991

**Published:** 2024-11-04

**Authors:** Abdul Hannan, Fiza Shaheen, Muhammad Saad

**Affiliations:** 1 Ophthalmology, Al-Shifa Trust Eye Hospital, Rawalpindi, PAK; 2 Ophthalmology, Shifa International Hospital Islamabad, Islamabad, PAK

**Keywords:** diabetic eye disease, pars plana vitrectomy, surgical instruments, type 2 diabetes mellitus, vitreoretinal diseases

## Abstract

Background

This study aimed to evaluate the safety, efficacy, and complications of 23-gauge (23-G) pars plana vitrectomy (PPV) in the treatment of various vitreoretinal diseases.

Methodology

A retrospective, cross-sectional study was conducted at Al-Shifa Trust Eye Hospital, Rawalpindi. All the eyes undergoing 23-G PPV between June 2020 and May 2023 meeting the inclusion criteria were included. Surgical outcomes and complications were assessed.

Results

A total of 350 eyes were included in our data. The most common indication was rhegmatogenous retinal detachment (RRD) (n = 144, 41.1%), followed by diabetic tractional retinal detachment (TRD) (n = 98, 21%). Satisfactory anatomical outcomes were achieved in all the indications. The primary success rate was 87.5% for RRD and 96.9% for TRD. The overall complication rate was 17.7%, with postoperative cataracts being the most frequent.

Conclusions

The 23-G PPV technique has proven to be a safe and effective approach for the surgical management of various vitreoretinal diseases, demonstrating favorable anatomical outcomes and a low rate of complications.

## Introduction

Pars plana vitrectomy (PPV) has undergone significant advancements over the years, evolving from 20-gauge systems to smaller gauge instruments such as 23-gauge (23-G), 25-gauge (25-G), and 27-gauge systems (27-G) [1]. These advancements have been pivotal in transforming the field of vitreoretinal surgery, offering benefits such as reduced operating time, quicker wound healing, and less postoperative inflammation [2].

In our region, where the introduction of the latest equipment and technology is often delayed, 23-G and 25-G systems are commonly available. The 25-G system, while effective, has certain limitations such as lower flow rates, increased instrument flexibility, and slightly longer operating times. As a result, it is typically used for less complex vitreoretinal conditions, as previously documented in the literature [3-5]. However, due to limited access to healthcare facilities and low awareness, we frequently encounter more complex vitreoretinal diseases, such as rhegmatogenous retinal detachments (RRDs) complicated by proliferative vitreoretinopathy (PVR) grade C and diabetic tractional retinal detachments (TRDs) with intricate membranes. Consequently, the 23-G system is predominantly used in our region. This study aims to report the anatomical outcomes and complication rates associated with the use of the 23-G PPV system in treating both simple and complex vitreoretinal diseases in our setting.

## Materials and methods

This retrospective, cross-sectional study was conducted at the vitreoretina department of Al-Shifa Trust Eye Hospital, Rawalpindi, after obtaining Institutional Review Board approval from Al-Shifa Trust Eye Hospital Research Center. Medical records of all patients who underwent 23-G PPV from June 01, 2020, to May 30, 2023, were retrospectively analyzed using the software system. The sample size of 350 eyes was calculated using OpenEpi with a 95% confidence level and 80% power. This number was refined based on available patient records to ensure adequate representation of various vitreoretinal diseases and surgical outcomes. Data of those patients who met the inclusion criteria was included. The study included eyes that underwent 23-gauge pars plana vitrectomy (PPV) for a range of vitreoretinal diseases, such as RRD, TRD, vitreous hemorrhage (VH), epiretinal membrane (ERM), macular hole (MH), and intraocular foreign body (IOFB). The study population consisted of patients aged between 18 and 80 years, with complete medical records available for analysis, which encompassed both preoperative and postoperative data up to three months after surgery. All surgeries were performed by a single vitreoretinal surgeon (AH) as it minimizes surgical bias and ensures consistency in surgical technique. Patients with a history of prior vitreoretinal surgery were excluded from the analysis. Assessed data variables included age, gender, eye laterality, and surgical indication, along with relevant peroperative details from the operative notes. The anatomical outcomes, complications if any, and further procedures if performed till three months from the operative date were also recorded. The primary outcome was defined as the surgical result after the first surgery while the final outcome was defined as the result after a further procedure or procedures if performed. Patients refusing further surgery were excluded from the final outcome.

The preferable mode of anesthesia was retro-bulbar block; however, in patients aged less than 20 years and in special circumstances according to the patient’s choice, it was replaced by general anesthesia. Eyes scheduled for simultaneous cataract extraction underwent phacoemulsification with intraocular lens implantation via a clear corneal incision of 2.75 mm after insertion of at least one trocar in the inferotemporal quadrant. The remaining two trocars were inserted afterward in the standard superonasal and superotemporal quadrants via an angled approach after turning on the infusion.

All the surgeries were done using the EVA phaco-vitrectomy system (DORC International, B.V.) using either of the three non-contact viewing systems, i.e., BIOM (Oculus Inc., Wetzlar, Germany), EIBOS (Haag-Streit surgical GmbH & Co. KG, Germany), and RESIGHT (Zeiss Medical Technology, Germany).

In all the cases, a core vitrectomy was done. Triamcinolone acetonide (0.2 mL of 40 mg/mL in 0.8 mL balanced salt solution) was used to stain the vitreous. Posterior vitreous detachment was induced if not already present, especially in eyes with RRD. This was followed by extensive base shaving with or without PVR peeling. In eyes with TRD, extensive membrane peeling was done using either or both of the 23-G serrated and end-gripping forceps. The dislocated intraocular lens was also removed with the 23-G serrated forceps through a corneal incision, followed by modified Yamane suture-less scleral fixation in eyes with healthy corneas. Dropped lens matter was removed entirely with a 23-G cutter; however, in eyes with dropped dense nuclei or nuclear fragments, a 20-G fragmotome was utilized. IOFB was also pulled out via 23-G serrated or end-gripping forceps. Internal limiting membrane (ILM) peeling was done in all the eyes with full-thickness macular hole (FTMH) using ILM blue dye (DORC International) and end-gripping 23-G forceps. At the completion, a 23-G endolaser barrage was done in all the eyes irrespective of diagnosis. In RRD, it was additionally done around breaks and retinotomy if created. In the case of diabetic TRD, pan-retinal photocoagulation was done. Cryotherapy was done in a few eyes with anteriorly located breaks. Peroperative tamponade (none, air, SF_6_ gas, C_2_F_6_ gas, C_3_F_8_ gas, silicon oil) was injected according to the surgical indication followed by the suturing of sclerotomies with 6-0 Vicryl in all the eyes. A subconjunctival injection comprising gentamycin and dexamethasone was given toward the end of the procedure.

SPSS version 21 (IBM Corp., Armonk, NY, USA) was used for data analysis. Qualitative measures were assessed through the calculation of frequencies and percentages, whereas for quantitative measures, mean and standard deviation were computed.

## Results

A total of 350 eyes were included in our study, comprising 230 (65.7%) male patients and 120 (34.3%) female patients. The mean age was 50.5 years ± 18.72 (mean ± standard deviation), with a slight predominance of the right eye (n = 179, 51.1%).

**Figure 1:**
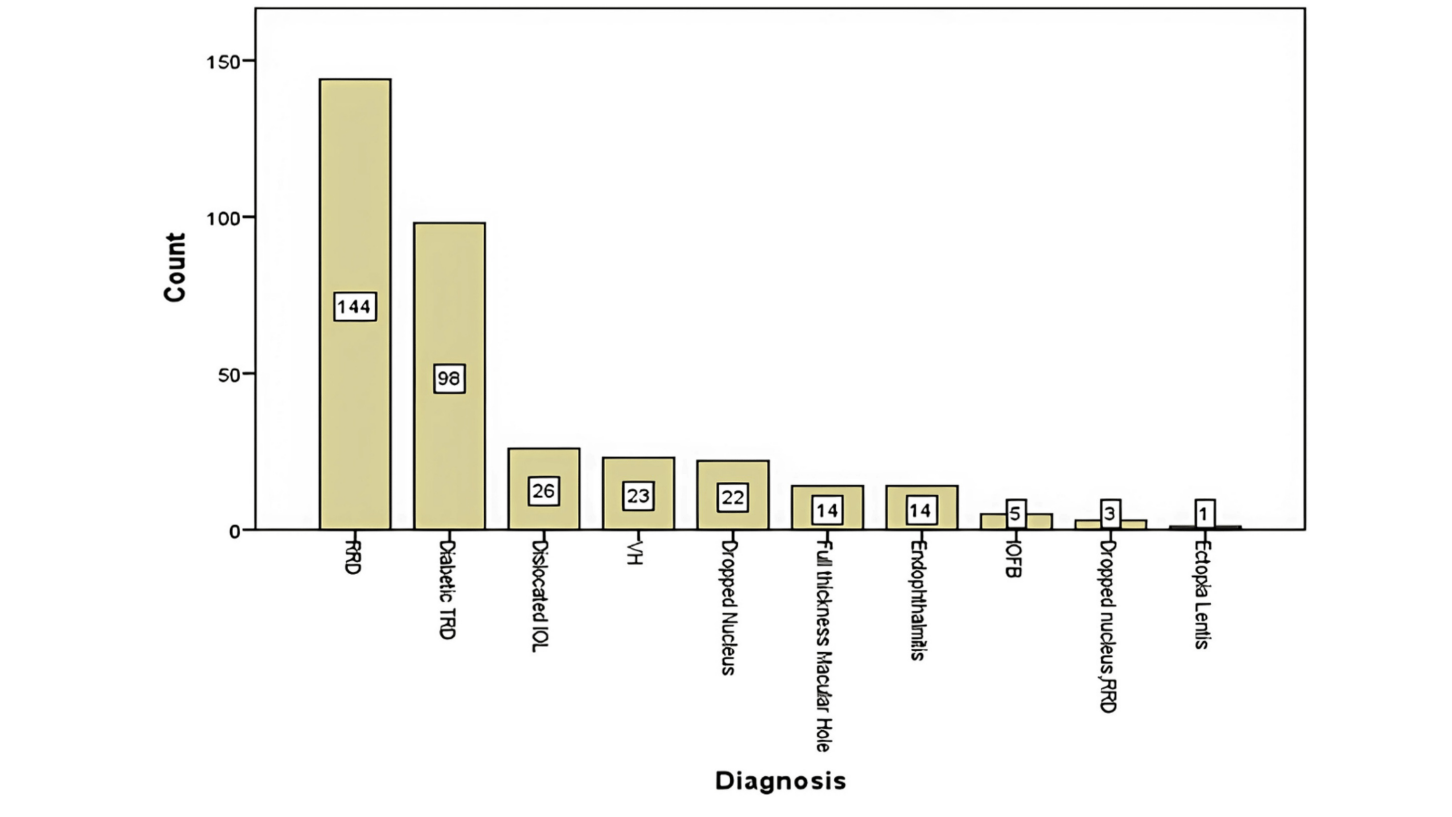
Each surgical indication along with the number of eyes. All values are presented as n unless otherwise specified. RRD = rhegmatogenous retinal detachment; TRD = tractional retinal detachment; IOL = intraocular lens; VH = vitreous hemorrhage; IOFB = intraocular foreign body

The majority of surgeries (n = 300), constituting 85.7% of cases, were performed under local anesthesia, suggesting its safety and patient tolerance, which aligns with current trends in ophthalmic surgery. The most common indication of surgery in our data was RRD (n = 144, 41.1%), followed by diabetic TRD (n = 98, 21%), reflecting the prevalence of these conditions in clinical practice. The rest of the diagnosis is presented in Figure 1.

Most of the eyes (n = 287), comprising 82% of cases, underwent isolated vitrectomy with specific maneuvers according to the diagnosis and disease level. A smaller proportion underwent combined phaco-vitrectomy (n = 43, 12.3%) or combined vitrectomy with scleral buckling (n = 20, 5.7%), particularly in RRD cases. Endolaser was the most commonly used peroperative technique (n = 298) applied in 85.1% of cases. Endolaser was consistently applied to reinforce retinal stability and reduce the risk of future complications. Cryopexy was used alone (n = 2, 0.6%) or in combination with laser (n = 6, 1.7%) in a few eyes. Silicon oil (5,000 cSt) was the most commonly used peroperative tamponade (n = 272, 77.7%).

Our results showed the primary success rate in RRD to be 87.5% (n = 126), with 12.5% (n = 18) of cases experiencing detachment. In diabetic TRD, the primary success rate was 96.9% (n = 95), with 3.1% (n = 3) of cases experiencing detachment. Primary outcomes of complex RRDs associated with IOFB and nucleus or IOL drop along with other indications are presented in Table 1.

**Table 1:** Diagnosis along with their respective outcomes. All values are presented as n and % unless otherwise specified. RRD = rhegmatogenous retinal detachment; PVR = proliferative vitreoretinopathy; SO = silicon oil; TRD = tractional retinal detachment; IOL = intraocular lens

Diagnosis	Primary outcome	Further surgery	Final outcome
RRD (n = 144)	Attached = 126 (87.5%); Detached = 18 (12.5%)	Inferior buckle = 10; PVR peel, SO = 5; Did not consent = 3	Attached = 15 (100%)
Diabetic TRD (n = 98)	Attached = 95 (96.9%); Detached = 3 (3.1%)	PVR peel, SO injection = 3	Attached = 3 (100%)
Dislocated IOL (n = 26)	Stable=26 (100%)	Nil	
Vitreous hemorrhage (n = 23)	Resolved = 23 (100%)	Nil	
Dropped nucleus (n = 22)	Stable = 22 (100%)	Nil	
Endophthalmitis (n = 14)	Resolved = 14 (100%)	Nil	
Full-thickness macular hole (n = 14)	Closed = 12 (85.7%); Open = 2 (14.3%)	Did not consent=2	
Intraocular foreign body (n = 5)	Attached = 4 (20%); Detached = 1 (80%)	Inferior buckle = 1	Attached = 1 (100%)
Dropped nucleus, RRD (n = 3)	Attached = 3 (100%)	Nil	
Ectopia lentis (n = 1)	Stable = 1 (100%)	Nil	

No anesthesia-related complications were noted in any of the eyes. The overall complication rate was 17.7% (n = 62), with cataracts being the most frequent complication (n = 30), occurring in 8.6% of cases. Other complications include raised IOL (n = 26, 7.4%) and iatrogenic retinal tears (n = 6, 1.7%). No cases of postoperative hypotony or wound leakage were observed in any of the eyes in this study.

## Discussion

Ever since the launch of the 23-G and 25-G and now the 27-G micro-incision vitrectomy system, use of the 20-G system is essentially replaced. While 25-G and 27-G are being adapted by many centers, we have evaluated the outcomes of 23-G PPV as there is still limited data available on its outcomes in our part of the world. In this retrospective study, we studied around 350 eyes that underwent 23-G PPV by the same vitreoretinal surgeon for multiple indications.

The most common indication in our data was RRD (n = 144, 41.1%) with all included eyes complicated by PVR grade C. The primary success rate was 87.5%, while the final success rate was 100% after excluding patients who refused further procedures. This is comparable to previous reports of 76.2% and 95.2% success rates with 23-G PPV [6], and considerably better than the 51% to 80% success rates reported with 20-G PPV [7-9]. Our rates are also comparable to the 77.8% and 92.6% success rates reported with 25-G PPV [10]. We found the 23-G instruments to be effectual for the extensive base shaving which is pivotal for the anatomical success in RRD. Akçay et al. [11] reported conversion to 20-G instrumentation for the removal of intense fibrovascular membranes. However, in our data, membrane peeling in both PVR-C complicated RRD and diabetic TRD was done successfully with 23-G instruments and none of the maneuvers required 20-G instrumentation. Our anatomical success rate of 96.9% and 100% in diabetic TRD is also noteworthy and consistent with the one reported before earlier 23-G [12] as well as with 27-G [13]. Anatomical outcomes reported with other indications were also satisfactory (Table 1). A list of previous studies with sample sizes and indications is presented in Table 2.

**Table 2:** Previous studies with sample sizes, indications, study designs, success rates, complication rates, and the exact interventions used. All values are presented as n unless otherwise specified. ERM = epiretinal membrane; VH = vitreous hemorrhage; FTMH = full-thickness macular hole; RRD = rhegmatogenous retinal detachment; TRD = tractional retinal detachment; VMT = vitreomacular traction; MIVS = micro-incision vitrectomy system

Study	Year of publication	Location	Sample size	Study design	Diagnosis	Exact intervention	Success rate (%)	Complication rate (%)
Gupta et al. [14]	2008	Multicenter (USA)	92	Retrospective, multicenter, interventional case series	ERM, VH, FTMH, RRD	23-gauge pars plana vitrectomy (PPV) using single-step entry system with trocar and cannula	Visual acuity improved from 20/238 to 20/82; 20/38 in macular hole cases (percentage not mentioned)	6.5% hypotony, 2.2% intraoperative retinal tear, 1.1% postoperative retinal tear, 1.1% recurrent retinal detachment
Schweitzer et al. [15]	2009	France	57	Prospective, non-comparative study	ERM, FTMH, diabetic retinopathy, RRD, silicon oil removal	23-gauge transconjunctival suture-less vitrectomy using two-step instrumentation	Improvement in mean visual acuity from 1.09 to 0.80 logMAR; 80% macular holes sealed	21.1% transient hypotony, 5.3% inflammation, minor conjunctival hemorrhage, no endophthalmitis
Akcay Bi et al. [11]	2011	Istanbul,Turkey	350	Prospective study	RRD, VH, TRD, FTMH, VMT, endophthalmitis, lens matter drop, vitreous opacities	23-gauge transconjunctival suture-less vitrectomy	Anatomical success in 86%, functional success in 72%	32% mild hypotony, 9.7% serious hypotony, 6.5% recurrent retinal detachment, 4.8% fibrinoid reaction, 22.5% cataract formation
Zas et al. [16]	2012	Argentina	75	Retrospective chart review	VH, RRD, ERM, FTMH, endophthalmitis	23-gauge transconjunctival suture-less PPV using two-step system	Statistically significant VA improvement in DVH, RRD, MEM, MH, endophthalmitis, BRVO, and PVR	Hypotony in 4%, recurrent pseudophakic retinal detachment in two cases
Cho et al. [17]	2011	New York, USA	14	Retrospective, non-comparative, interventional case series	Posteriorly dislocated crystalline lens	23-gauge PPV with optional 20-gauge fragmatome for dense lens fragments	57% achieved 20/40 vision or better	14% choroidal detachment, 14% vitreous hemorrhage, 14% ocular hypertension
Figueroa et al. [18]	2015	Multicenter (Spain)	30	Prospective, multicenter, non-randomized study	Myopic traction maculopathy without macular hole or retinal detachment	23-gauge PPV with internal limiting membrane (ILM) peeling stained with brilliant blue G, and gas tamponade (12% C3F8)	93% MTM resolution; significant BCVA improvement in 60% of eyes	3% macular hole, 6.7% retinal detachment, 40% nuclear cataracts
Chan et al. [19]	2020	London, UK	291	Retrospective, interventional case series	Retained lens fragments after complicated cataract surgery	23-gauge MIVS with additional use of 20-gauge phacofragmatome in 63.9% cases	62.9% achieved BCVA of 20/40 or better	10% ocular hypertension, 8.6% transient CME, 3.1% retinal detachment, 2.7% persistent corneal edema, 1.4% macular holes

Even with the advent of 25-G and 27-G instrumentation, several surgeons still opt for the 23 G system as it delivers all the benefits of the 25-G system while having superior fluid dynamics, better suction, an enhanced central vitrectomy, and smoother peripheral retinal manipulation along with a quicker silicone oil injection [12]. Additionally, there are still technical issues with 25-G and 27-G PPV, including more challenging instrument handling with thin and flexible tools, vision quality limitations due to the limited endo-illumination, and more challenging silicone oil tamponade [20,21]. Finally, the choice of surgery is influenced by the equipment’s accessibility, the case’s complexity, and the surgeon’s preferences.

While 23-G PPV has become a standard in our region due to its balance of rigidity and fluid dynamics, emerging evidence on smaller gauge systems, particularly 25-G and 27-G, highlights some comparative benefits and limitations. Although the 25-G and 27-G systems reduce incision size and enhance patient comfort through faster recovery, these gauges exhibit increased flexibility, which can hinder precise manipulation in dense or complex vitreoretinal cases. In the literature, one of the main disadvantages of 23-G PPV is postoperative hypotony [12,22]. Kusuhara et al. [22] reported a hypotony rate of 1.5% while a local study [23] reported it to be 10.77%. Shimada et al. [24] reported postoperative hypotony more in the fluid-filled cavity compared to gas or air-filled cavity with 23-G system. Notably, there were no observed cases of postoperative hypotony associated with any type of tamponade used in this study. According to Eckardt [25], the incision angle is more significant than its size in preventing postoperative hypotony. We found that both the angle as well as suturing of wounds is crucial to prevent unfavorable complications. In a retrospective case study, Kunimoto et al. [26] found that the incidence of endophthalmitis following sutureless 25-G PPV is 12 times greater than that following standard 20-G PPV. Although suturing prolongs the surgery by a few minutes and is prone to cause irritation [27], we found that the risk of wound leak, hypotony, choroidal detachment, and endophthalmitis is essentially reduced to zero with sutured sclerotimes. These findings are especially relevant in our part of the world where patient care and hygiene standards may pose unique challenges, making the prevention of complications even more critical.

The main limitation of our study is shorter follow up time period and absence of visual outcomes. We propose future larger prospective studies including both the visual and anatomical outcomes with the 23-G PPV system.

## Conclusions

Our study demonstrated that 23-G vitrectomy is effective for managing various retinal conditions. Most RRD cases were successfully reattached, though some needed additional procedures such as inferior buckle and silicone oil. Diabetic TRD was well-managed without further surgery. Dislocated IOLs, VH, dropped nucleus, and endophthalmitis resolved or stabilized without additional interventions. Full-thickness macular holes showed significant closure, and IOFBs were successfully addressed with further treatment. Complications included mild-to-moderate inflammation, a few infections, and instances of recurrent detachment. These results highlight the success of 23-G vitrectomy and the need for diligent postoperative monitoring.
